# Durable responses to alectinib in murine models of EML4-ALK lung cancer requires adaptive immunity

**DOI:** 10.1038/s41698-023-00355-2

**Published:** 2023-02-04

**Authors:** Emily K. Kleczko, Trista K. Hinz, Teresa T. Nguyen, Natalia J. Gurule, Andre Navarro, Anh T. Le, Amber M. Johnson, Jeff Kwak, Diana I. Polhac, Eric T. Clambey, Mary Weiser-Evans, Daniel T. Merrick, Michael C. Yang, Tejas Patil, Erin L. Schenk, Lynn E. Heasley, Raphael A. Nemenoff

**Affiliations:** 1 Department of Medicine, University of Colorado Anschutz Medical Campus, Aurora, CO USA; 2 Department of Craniofacial Biology, University of Colorado Anschutz Medical Campus, Aurora, CO USA; 3 Eastern Colorado VA Healthcare System, Rocky Mountain Regional VA Medical Center, Aurora, Colorado USA; 4 Department of Anesthesiology, University of Colorado Anschutz Medical Campus, Aurora, CO USA; 5 Department of Pathology, University of Colorado Anschutz Medical Campus, Aurora, CO USA

**Keywords:** Cancer microenvironment, Non-small-cell lung cancer

## Abstract

Lung cancers bearing oncogenic EML4-ALK fusions respond to targeted tyrosine kinase inhibitors (TKIs; e.g., alectinib), with variation in the degree of shrinkage and duration of treatment (DOT). However, factors that control this response are not well understood. While the contribution of the immune system in mediating the response to immunotherapy has been extensively investigated, less is known regarding the contribution of immunity to TKI therapeutic responses. We previously demonstrated a positive association of a TKI-induced interferon gamma (IFNγ) transcriptional response with DOT in EGFR-mutant lung cancers. Herein, we used three murine models of EML4-ALK lung cancer to test the role for host immunity in the alectinib therapeutic response. The cell lines (EA1, EA2, EA3) were propagated orthotopically in the lungs of immunocompetent and immunodeficient mice and treated with alectinib. Tumor volumes were serially measured by μCT and immune cell content was measured by flow cytometry and multispectral immunofluorescence. Transcriptional responses to alectinib were assessed by RNAseq and secreted chemokines were measured by ELISA. All cell lines were similarly sensitive to alectinib in vitro and as orthotopic tumors in immunocompetent mice, exhibited durable shrinkage. However, in immunodeficient mice, all tumor models rapidly progressed on TKI therapy. In immunocompetent mice, EA2 tumors exhibited a complete response, whereas EA1 and EA3 tumors retained residual disease that rapidly progressed upon termination of TKI treatment. Prior to treatment, EA2 tumors had greater numbers of CD8+ T cells and fewer neutrophils compared to EA1 tumors. Also, RNAseq of cancer cells recovered from untreated tumors revealed elevated levels of CXCL9 and 10 in EA2 tumors, and higher levels of CXCL1 and 2 in EA1 tumors. Analysis of pre-treatment patient biopsies from ALK+ tumors revealed an association of neutrophil content with shorter time to progression. Combined, these data support a role for adaptive immunity in durability of TKI responses and demonstrate that the immune cell composition of the tumor microenvironment is predictive of response to alectinib therapy.

## Introduction

Lung adenocarcinomas (LUADs) driven by oncogenic tyrosine kinases including mutant EGFR and ALK fusions exhibit frequent and extensive responses to precision targeted tyrosine kinase inhibitors (TKIs), although with a wide range in the depth of response (DepOR) and time to progression or duration of treatment (DOT)^1–3^. Alectinib, the current first-line standard of care for metastatic ALK+ lung cancers, yields objective tumor responses (<30% tumor shrinkage by RECIST) in ~50–75% of TKI-treated patients and progression free survival (PFS) ranges from ~10 to 30 months. Despite these successes, TKIs fail to completely eliminate tumor cells, with remaining cancer cells referred to as “drug tolerant persisters”^4^ or “residual disease”^5^. An association has been reported between initial DepOR and progression free and overall survival in ALK+ patients^6^. Thus, understanding the biological underpinnings for variation in DepOR and time to progression may inform rational strategies for enhancing the efficacy of oncogene-targeted agents through novel drug combinations. Our recent published studies demonstrate that EGFR-targeted inhibitors induce an interferon (IFN) response program that varies markedly between distinct EGFR mutant lung cancer cell lines and positively associates with the duration of therapeutic response in EGFR-mutant lung cancer patients^7^. In fact, there is a growing literature supporting the role of host immune cells in overall therapeutic response to precision oncology agents and cytotoxic drugs^8–19^. However, the mechanisms whereby TKIs induce factors mediating paracrine signaling to the immune microenvironment, and the contribution to the overall therapeutic response are not well understood. This is critical to understanding how these pathways may be targeted for therapeutic gain.

While human samples can be used to develop correlations, preclinical mouse models that recapitulate critical features of the human disease open avenues to deep mechanistic exploration. To define the role of the tumor microenvironment (TME) in mediating response to TKI therapy in ALK positive lung cancer, we have used an orthotopic immunocompetent model whereby murine EML4-ALK fusion-positive lung cancer cells are directly implanted into the lungs of syngeneic C57BL/6 mice^20–22^. In contrast to studies that have implanted lung cancer cell lines subcutaneously, this model requires tumors to develop in the relevant microenvironment of the lung such that the role of the adaptive and innate immune systems in contributing to the overall therapeutic response can be assessed. By examining a panel of cell lines with the same oncogenic driver, we sought to model the heterogeneity of response observed in patients. In this study, we demonstrate a critical role for adaptive immune cells as contributors to the response to ALK TKI therapy. Furthermore, when propagated in immunocompetent mice, distinct ALK-driven lung cancer cells exhibit differences in the depth and duration of response to the ALK inhibitor, alectinib which correlate with the composition of the immune microenvironment. In addition, we translated these findings into human ALK+ patients through analysis of pre-treatment biopsy specimens.

## Results

### Therapeutic response of primary EML4-ALK tumors to ALK-targeting TKIs

Maddalo et al.^23^ demonstrated that intratracheal delivery of an adenovirus encoding Cas9 and gRNAs targeting murine *Eml4* and *Alk* yielded efficient generation of EML4-ALK positive murine lung tumors. Likewise, we observed that instillation of Adeno-Cas9-gRNA virus induces multiple tumor foci in C57BL/6 mice (Supplementary Fig. 1). Although limited by the sensitivity of μCT as a detection method, the vast majority of lesions underwent rapid and significant shrinkage following alectinib treatment as initially noted^23^. Moreover, TKI treatment for 4 weeks and then termination of therapy resulted in prompt regrowth of some, but not all, of the lesions within 4 weeks, indicating the presence of residual disease. Thus, this murine model of EML4-ALK-driven lung cancer replicates the heterogeneity of response to therapy, a key feature of the human disease. For further investigations of the molecular mechanisms mediating variation in the depth and duration of response, we developed a panel of transplantable murine EML4-ALK cancer cell lines derived from this model.

### Murine EML4-ALK positive lung cancer cell lines exhibit equivalent in vitro sensitivity to TKIs, but distinct therapeutic responses in an orthotopic model

**Fig. 1:**
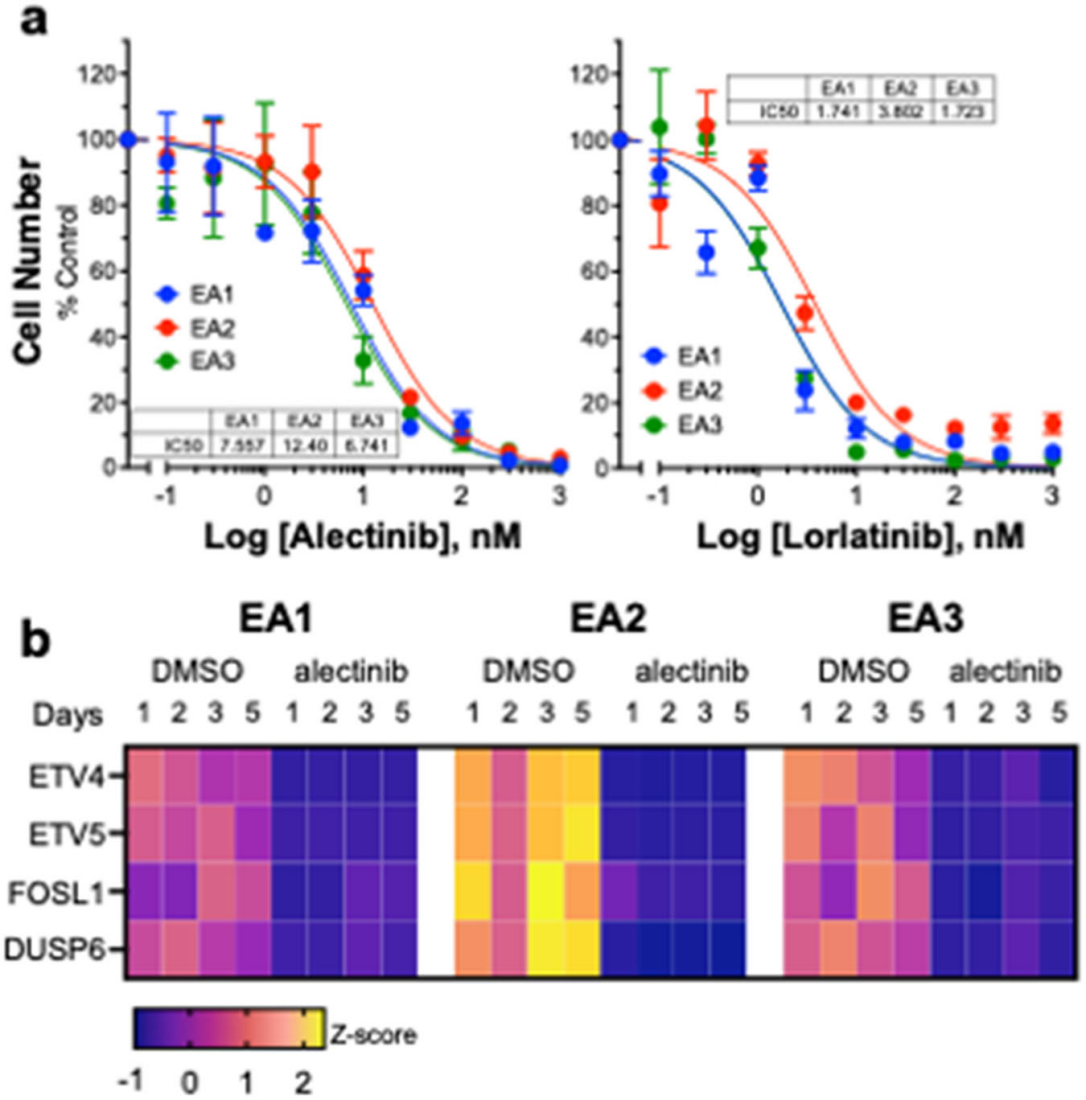
Murine EML4-ALK cell lines exhibit equal in vitro sensitivity to TKIs. **a** EA1, EA2, and EA3 cells were seeded at 100 cells/well in 96-well plates and cultured in growth medium containing the indicated concentrations of the TKIs, alectinib or lorlatinib. After 7 days of incubation, cell number was determined using the CyQUANT assay (see “Methods”). Data (mean and SEM of triplicate determinations) are presented as percent of wells treated with medium containing 0.1% DMSO. The IC_50_ values for alectinib (7.6, 12.4, and 6.7 nM for EA1, EA2, and EA3, respectively) and lorlatinib (1.7, 3.8, and 1.7 nM for EA1, EA2, and EA3, respectively) were calculated with Prism 9. **b** RNAseq data from the murine EML4-ALK cell lines treated with and without alectinib (see “Methods”) were queried for known MAPK target genes, ETV4, ETV5, FOSL1, and DUSP6. The mRNA expression values (CPMs) were converted to *Z*-scores and presented as a heatmap.

A murine ALK+ cell line referred to herein as EA2 is a generous gift of Dr. Andrea Ventura^23^. We also developed two additional EML4-ALK-driven cell lines, EA1^24^ and EA3 within our institution. All cell lines were isolated from C57BL/6 mice and express the mRNA encoding the EML4-ALK variant 1 fusion gene compared to KRAS-mutant murine LLC lung cancer cells (Supplementary Fig. 2a, b). EA2 and EA3 cell lines were derived from TP53 null mice while EA1 was derived from a TP53 wild-type mouse. The immunoblot in Supplementary Fig. 2c confirms TP53 protein expression in EA1, but not EA2 or EA3 cells. The three cell lines exhibited equivalent in vitro growth sensitivity to the ALK TKIs, alectinib and lorlatinib (Fig. 1a). In addition, the findings in Fig. 1b show equivalent reduction of mRNA levels of the MAPK pathway-regulated genes, ETV4, ETV5, FOSL1 and DUSP6^25,26^ by alectinib. Thus, these data demonstrate equal alectinib sensitivity of EA1, EA2 and EA3 cells using functional in vitro assays.

### Adaptive immunity is required for durable alectinib responses

**Fig. 2:**
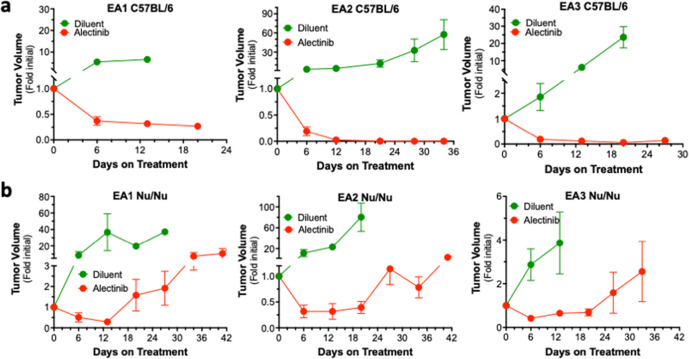
Alectinib responsiveness of orthotopically-propagated EML4-ALK tumors. **a** The indicated murine EML4-ALK cell lines (500,000 cells per mouse) were implanted into the left lungs of C57BL/6 mice. The orthotopic tumors established for ~10 days, the mice were submitted to μCT imaging to determine pre-treatment tumor volumes and following randomization, treated daily with 20 mg/kg alectinib or diluent by oral gavage. Mice were imaged by µCT weekly over the course of the experiments and tumor volume is presented as the fold change from the initial pre-treatment measurement. The initial tumor volumes (mean ± SEM) for the diluent and alectinib-treated groups were 8.5 ± 7.9 and 11.3 ± 9.1 mm^3^, 10.9 ± 8.5 and 11.6 ± 8.8 mm^3^, and 2.6 ± 2.5 and 1.9 ± 1.6 mm^3^ for EA1, EA2, and EA3, respectively. **b** The cell lines were implanted into the left lungs of *nu/nu* mice, and mice were treated as described in **a**. Tumor volumes are presented as the fold change from the initial pre-treatment measurement. The initial tumor volumes (mean ± SEM) for the diluent and alectinib-treated groups were 3.8 ± 2.6 and 4.2 ± 3.7 mm^3^, 8.7 ± 10.5 and 9.4 ± 11.2 mm^3^, and 18.9 ± 11.6 and 20.5 ± 13.5 mm^3^ for EA1, EA2, and EA3, respectively.

**Fig. 3:**
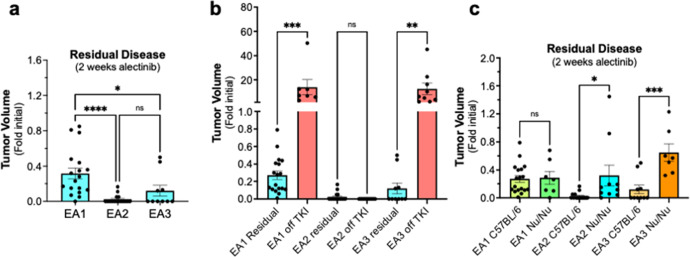
Distinct DepOR among EML4-ALK models and adaptive immunity requirement for maximal DepOR. **a** The tumor volumes (fold change from initial) after 2 weeks of alectinib treatment in the EA models (see Fig. 2a) were analyzed by one-way ANOVA (Kruskal–Wallis test). **b** Tumor-bearing mice treated for 3 weeks with alectinib (Residual, from Fig. 2) were followed by μCT for another 2 weeks after termination of daily TKI treatment. The tumor volumes relative to initial pre-treatment volumes are presented and the data were analyzed by one-way ANOVA. **c** The residual disease volumes measured after 2 weeks of alectinib treatment in C57BL/6 were compared to *Nu/Nu* hosts by two-tail *t* tests. In the graphs, the error bars are the standard error of the mean and dots represent individual tumors. *, **, ***, and **** indicate *p* values <0.05, <0.01, <0.001 and <0.0001 respectively.

The three EML4-ALK cell lines were used to establish orthotopic tumors in the left lung lobe of syngeneic C57BL/6 mice (see “Methods” and refs. ^20,22,27,28^). Following tumor establishment for ~10–14 days, the initial tumor volume was measured by μCT and the mice were treated daily with alectinib (20 mg/kg) or diluent by oral gavage and tumor volumes were measured weekly. Marked tumor shrinkage was induced by alectinib in all three models (Fig. 2a). Close inspection of the alectinib responses reveals distinct degrees of maximal DepOR following 2 weeks of treatment as assessed by μCT imaging (Fig. 3a). The DepOR was greatest in EA2 followed by EA3 with the least shrinkage in EA1 tumors. When daily alectinib treatment was terminated after 3 weeks, tumors rapidly progressed in the EA1 and EA3 tumor-bearing mice where residual disease was evident, but not in EA2-bearing mice (Fig. 3b). Thus, 3–4 weeks of TKI treatment yields variable degrees of viable residual disease.

In our previous study, pan-ERBB TKI response in orthotopic murine head and neck tumors was diminished in immune-deficient hosts^29^. To test the role of the adaptive immune system in the alectinib therapeutic response, the murine EML4-ALK cells were inoculated into the left lungs of *nu/nu* mice lacking functional T and B cells. In all three EML4-ALK cell line models, significant tumor shrinkage was elicited by alectinib, but was followed by prompt tumor progression within three to four weeks of initiating treatment, despite continuous TKI therapy (Fig. 2b). EA2 cells were also inoculated into the orthotopic site of immune-deficient Rag1^−/−^ mice and the resulting tumor-bearing mice were treated with diluent or alectinib. Similar to *nu/nu* mice, transient tumor shrinkage was observed, but tumor progression occurred within 3 weeks of initiating treatment (Supplementary Fig. 3a). Combined, these data indicate that functional adaptive immune cells are critical for the sustained anti-cancer activity of alectinib observed in C57BL/6 mice. Comparison of the degree of shrinkage in C57BL/6 hosts versus *nu/nu* mice revealed greater shrinkage with EA2 and EA3 tumors in immunocompetent mice, whereas no difference in degree of alectinib-induced shrinkage was observed in EA1 tumors propagated in C57BL/6 vs. *nu/nu* hosts (Fig. 3c). Thus, adaptive immunity contributes to both the depth and the durability of alectinib response.

### Analysis of the immune microenvironment in murine EML4-ALK tumors

**Fig. 4:**
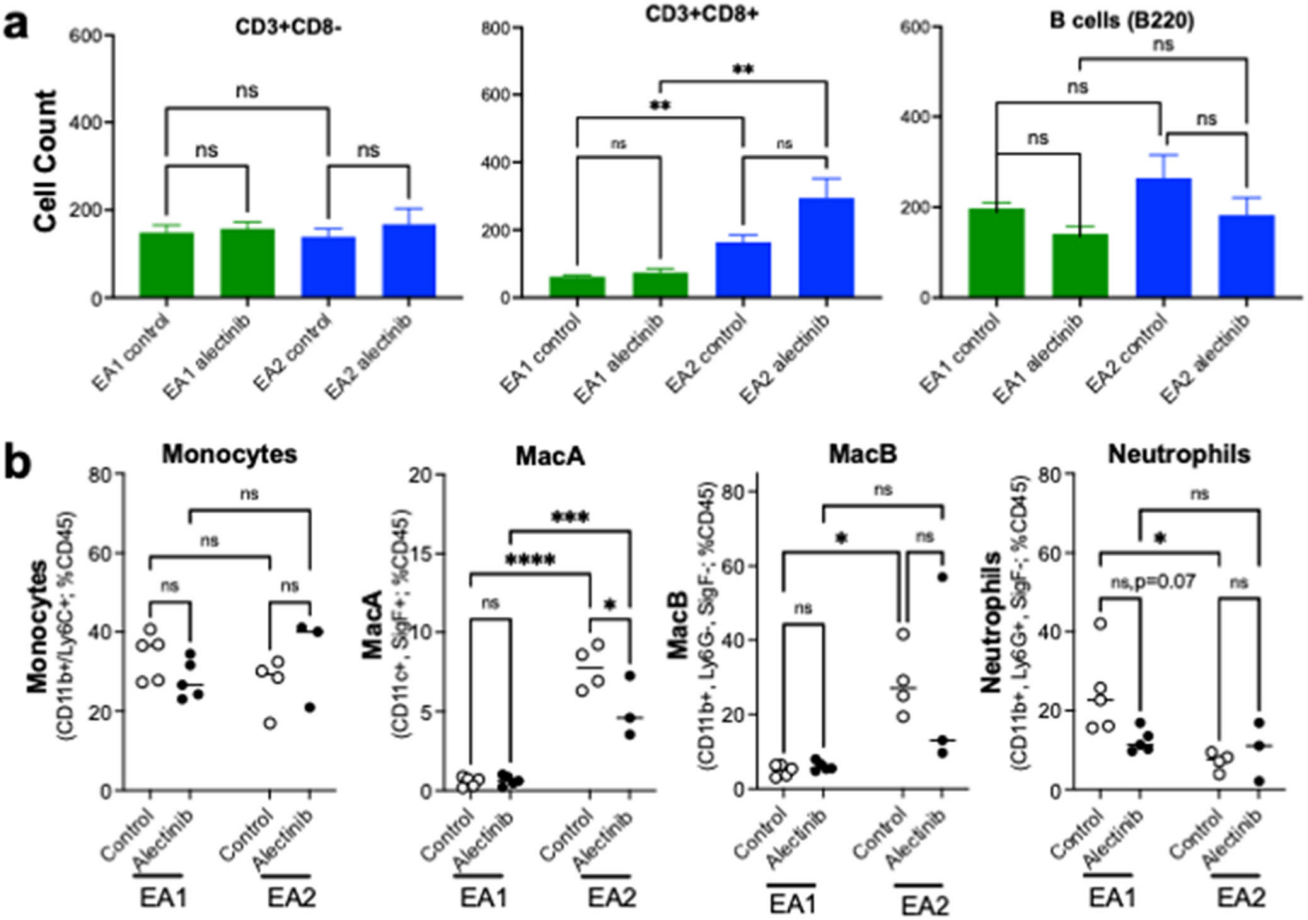
Adaptive and innate immune cells in control and alectinib-treated EA1 and EA2 tumors. EA1 or EA2 cells were implanted in the left lung of C57BL/6 mice. Tumors were established for 3 weeks and then mice were treated for 4 days with either control or 20 mg/kg alectinib. **a** Tissue was harvested, processed, and FFPE slides were submitted to multispectral imaging using Vectra Polaris. Data acquisition, segmentation, and cell phenotyping was performed using inForm software. The R package Akoya Biosciences phenoptrReports was used to count CD3+/CD8+ and CD3+/CD8− cells (presumed to be CD4+ T cells) as well as B220-positive B cells. Differences between groups was analyzed by the Kruskal-Wallis test with correction for multiple comparisons. **b** After 4 days of treatment, mice were sacrificed and a single-cell suspension was made from the left tumor-bearing lung. The single cell suspension was stained and submitted to flow cytometry analysis for innate immune cells using the previously described gating strategy^30^. Differences between groups were analyzed with ordinary one-way ANOVA and correction for multiple comparisons. Data are presented as the mean ± SEM where *, **, ***, and **** indicate *p* values <0.05, <0/01, <0.001, and <0.0001, respectively.

The findings in Figs. 2 and 3 support a role for the tumor microenvironment in mediating the response to TKI therapy. To analyze the immune cell populations in control and alectinib-treated tumors, a combination of multispectral imaging (Polaris Vectra) and standard flow cytometry was deployed with the EA1 model which exhibits a partial response to alectinib and the EA2 model that exhibits a complete response. EA1 and EA2 cells were implanted into mice and allowed to establish for 3 weeks and then treated for 4 days with control or 20 mg/kg alectinib. The left lungs of C57BL/6 mice bearing established EA1 and EA2 tumors were harvested, formalin-fixed and submitted to Polaris Vectra imaging with a panel of antibodies targeting immune cells (see “Methods”). We focused on CD8+ T cells, as defined as CD3+/CD8+ positivity, and the CD3+/CD8− phenotype identifying CD4+ T cells. Also, B cells were assessed by positivity for B220 staining. In untreated tumors, significantly greater numbers of tumor-infiltrated CD8+ cells were observed in EA2 tumors compared to EA1, with comparable numbers of CD3+ CD8− cells (Fig. 4a). Following 4 days of alectinib treatment, EA2 tumors exhibited a trend towards further increase in CD8+ T cells, although not statistically significant, while EA1 tumors showed no change in CD8+ or CD4+ T cell content with treatment. The content of B cells within control EA1 and EA2 tumors was similar with no evidence of alterations in response to a 4-day alectinib treatment (Fig. 4a).

To identify potential changes in myeloid cell types, we submitted dissociated tumor-bearing lungs to flow cytometric analysis using a previously reported gating strategy^30^. No differences in monocyte (defined as CD11b+/Ly6C+) content was observed between EA1 and EA2 tumor-bearing lungs harvested from control or alectinib-treated mice (Fig. 4b). Control EA2 tumors exhibited increased content of alveolar macrophages (MacA: defined as CD11c+/SigF+) and recruited monocyte/macrophage populations (MacB: defined as CD11b+/Ly6G−/SigF−)^30^ compared to EA1 tumors and the content of MacA cells was reduced following a 4-day alectinib treatment (Fig. 4b). By contrast, control EA1 tumors had elevated neutrophil (defined as CD11b+/Ly6G+/SigF−) content compared to EA2 tumors and alectinib induced a decrease in the neutrophil population that approached statistical significance (Fig. 4b).

### Alectinib induces an IFNγ-like transcriptional program and varied expression of distinct chemokines

Our recent studies in EGFR mutant lung cancer cell lines and murine head and neck cancer cells demonstrated a TKI-stimulated IFNγ transcriptional response accompanied by increased chemokine expression^7,29^. The three murine EML4-ALK cell lines were treated in vitro with alectinib or DMSO and RNAseq was performed (see “Methods”). The resulting datasets were submitted to gene-set enrichment analysis (GSEA) using the Hallmark Pathways and the normalized enrichment scores (NES) are presented as a heatmap in Supplementary Fig. 4. The data show that multiple inflammation-related Hallmark pathways are enriched in alectinib-treated cells including IFNα, IFNγ, allograft rejection and inflammatory response. In addition, Hallmark pathways associated with cell proliferation (MYC targets V1 and V2, G2M checkpoint, E2F targets) are markedly de-enriched in the alectinib-treated cells and provides further support for equivalent growth inhibition by alectinib in the three cell lines.

**Fig. 5:**
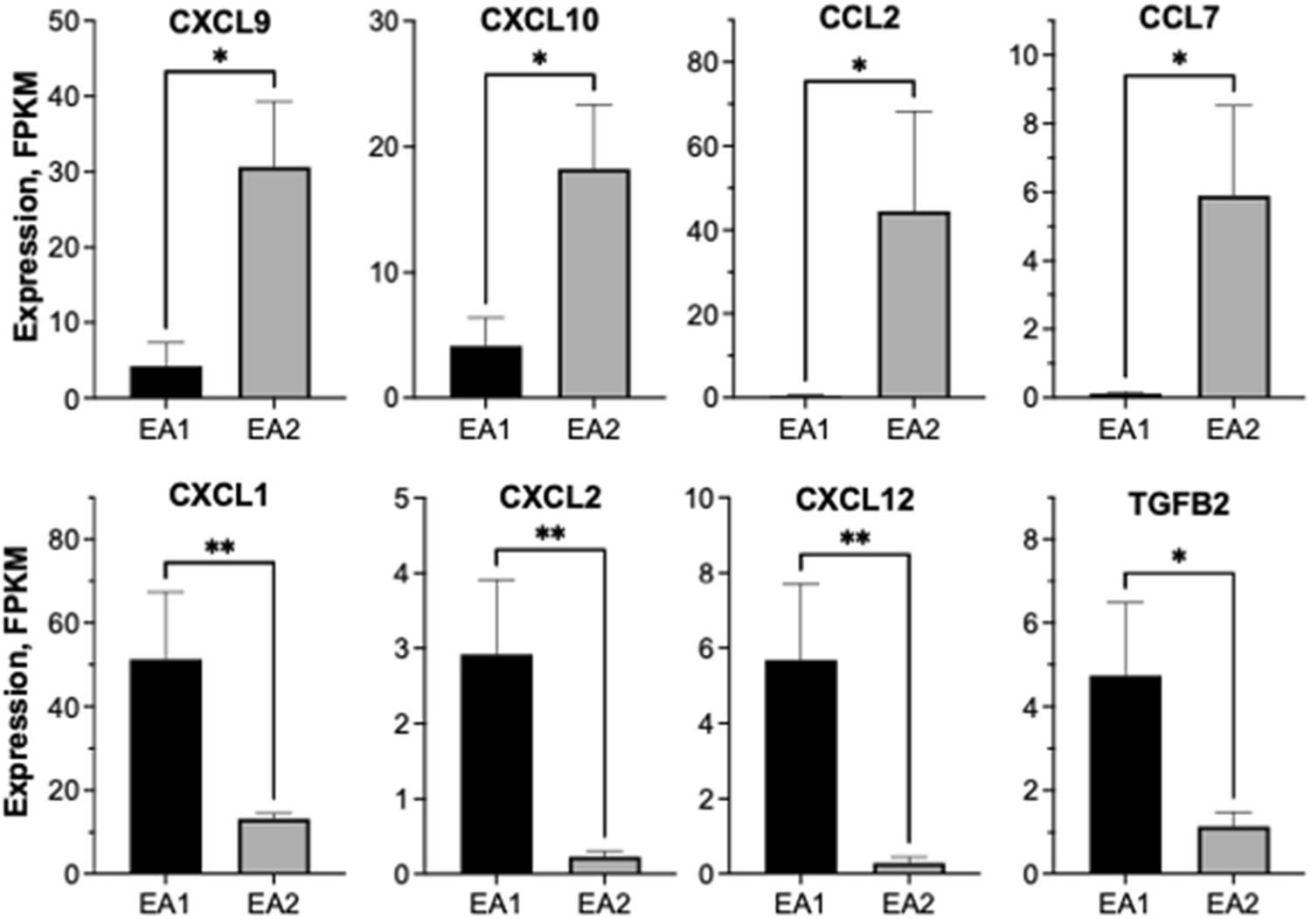
Distinct baseline chemokine expression patterns in EA1 and EA2 orthotopic tumors. EA1 and EA2 tumors were established in C57BL/6 mice in which GFP was ubiquitously-expressed in all cell types (see “Methods”). After ~3 weeks, the left lung lobes from the tumor bearing mice (*n* = 5 for each cell line) were dissociated into single-cell suspensions and submitted to flow sorting to collect the GFP-negative tumor cells. RNA was purified from the sorted cells and submitted to RNAseq. The data show the mRNA expression in FPKM (mean and SEM) where * and ** indicate *p* values <0.05 and <0.01, respectively.

To interrogate diversity of chemokine expression amongst the EML4-ALK lines when propagated as orthotopic tumors, the cancer cells from untreated EA1 and EA2 tumors were purified from transgenic GFP-expressing mice and submitted to RNAseq analysis (see “Methods”). As shown in Fig. 5, mRNA levels of CXCL9 and CXCL10, two chemokines with T cell recruitment functions, were significantly higher in EA2 tumors compared to EA1. Expression of CCL2 and CCL7 expression was also higher in untreated EA2 relative to EA1 tumors. By contrast, levels of CXCL1, CXCL2, CXCL12 and TGF-β2, chemokines and cytokines associated with recruiting immunosuppressive populations^18,31,32^ were higher in EA1 tumors relative to EA2 tumors.

**Fig. 6:**
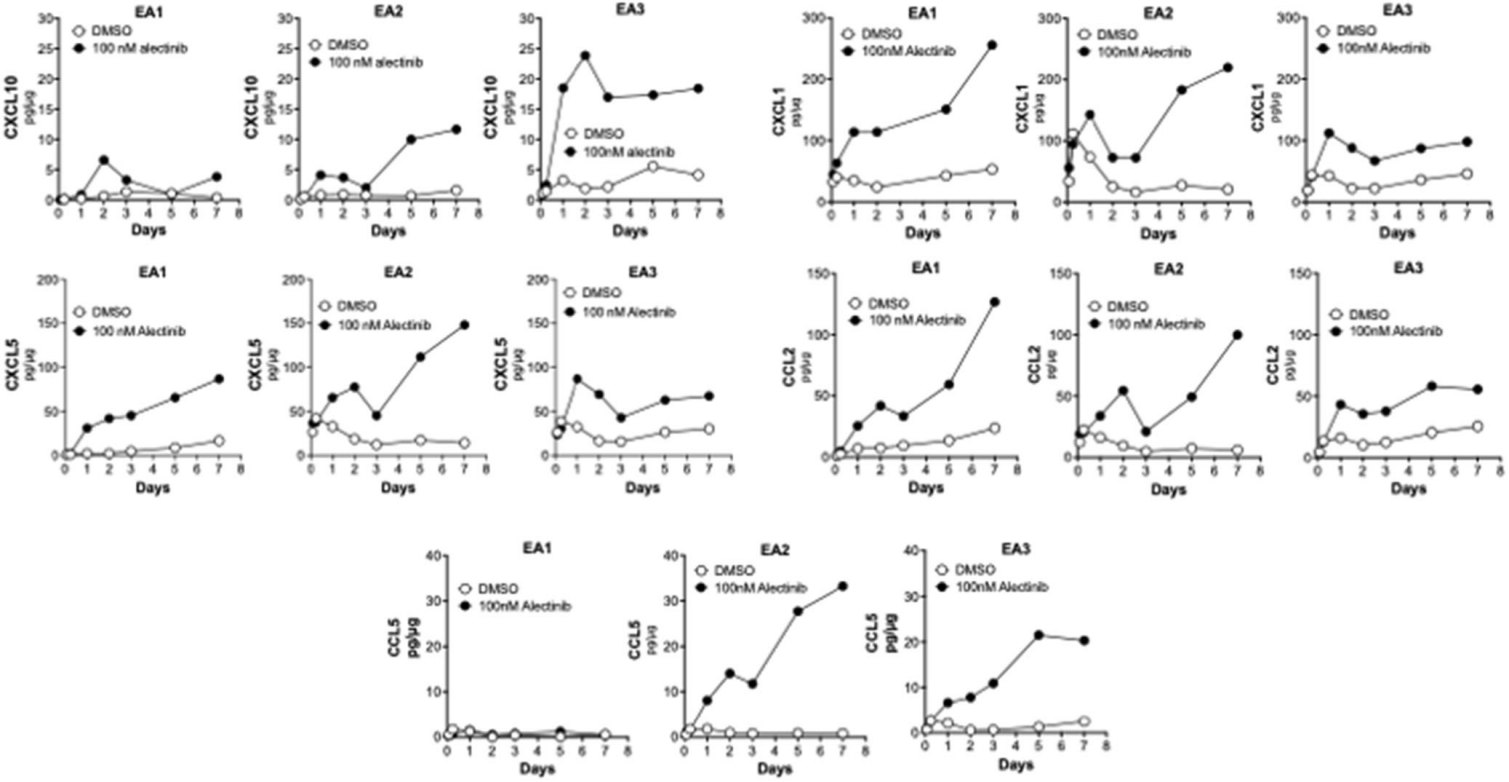
Chemokine induction with alectinib treatment in murine EML4-ALK cell lines. EA1, EA2, and EA3 cells were treated in vitro with 0.1% DMSO or 100 nM alectinib for 2 h to 7 days. The culture medium was collected and submitted to ELISA for the indicated chemokines. The data are presented as pg of analyte per μg cell protein and are representative of two independent experiments.

Specific chemokines and cytokines are central to the genes comprising the inflammation-related Hallmark pathways. Using the RNAseq data as a guide, multiple chemokine genes were selected and the effect of alectinib treatment on their secretion was determined by ELISA. The findings in Fig. 6 reveal alectinib-stimulated secretion of multiple chemokines with diverse activities as anti- and pro-tumorigenic factors. CXCL10 and CCL5 were markedly induced in response to alectinib in EA2 and EA3 cells, but weakly in EA1 cells. By contrast, alectinib-induced secretion of CXCL1, CXCL5 and CCL2 with roles in the recruitment of lymphocytes of myeloid lineages were similar among the three cell lines. In general, the level of chemokine expression measured in vitro with untreated cell lines was very low, indicating that signals from the murine hosts are distinctly integrated by EA1 and EA2 cells to achieve the distinct levels observed in vivo.

### Quantification of lymphocytes and PMNs in ALK+ patient biopsies

**Fig. 7:**
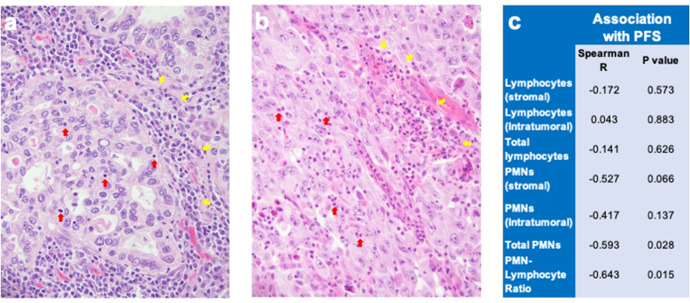
Lymphocytes and polymorphonuclear neutrophil (PMN) quantification in ALK+ patient pre-treatment biopsies and correlation with PFS. Representative images are shown of H&E-stained pre-treatment biopsies from ALK+ lung cancer patients indicating tumor-infiltrating lymphocytes (**a**) and PMNs (**b**). In **a**, tumor-infiltrating lymphocytes (LCs) including tumor stromal LCs (yellow arrows) and intratumoral LCs (red arrows) are indicated. In **b**, tumor-infiltrating PMNs including tumor stromal PMNs (yellow arrows) and intratumoral PMNs (red arrows) are shown. **c** Spearman correlation analysis of PFS with pre-treatment tumor content of lymphocytes and PMNs and the PMN/lymphocyte ratio is tabulated.

To explore associations of the host immune microenvironment with ALK-targeting TKI response in patients, H&E-stained sections of biopsy specimens obtained at the time of LUAD diagnosis, prior to TKI treatment were obtained from 14 ALK+ patients. All eventually progressed on first-line TKI therapy and PFS ranged from 1-54 months (mean and median = 14.2 and 9 months, respectively). Lymphocytes and PMNs (both characterized by their distinct nuclear morphology) were quantified in the tumor and stromal compartments. Representative images of tumor infiltrating lymphocytes (TILs) and PMNs are shown in Fig. 7a, b. The resulting values were submitted to Spearman correlation with the patient’s PFS. The PFS negatively associated with the total content of PMNs (*R* = −0.593, *p* = 0.028) and the ratio of total PMNs to total lymphocytes (*R* = −0.643, *p* = 0.015), but not stromal, intratumoral or total lymphocyte content (Fig. 7c). The findings indicate that high baseline PMN content is associated with reduced duration of TKI response.

## Discussion

EML4-ALK lung cancers possess a low mutational burden and patients exhibit little or no response to anti-PD-1 checkpoint inhibitor therapy. Rather,these patients are generally treated with TKIs such as alectinib, that selectively target the oncogenic driver. Relevant to the studies presented herein, the ineffectiveness of immune checkpoint inhibitors should not be interpreted as a lack of involvement of host immunity in the therapeutic response to TKIs. In fact, emerging evidence indicates that the tumor microenvironment is not a passive bystander, but is a determinant of the depth and duration of response. Cancer cell death in response to ALK-specific TKIs has been shown to be immunogenic^11,16^. In fact, it has become apparent that contribution from the TME is critical to the response to oncogene-targeted agents in general^16,17^. For example, CDK4/6 inhibitors induce cytokines that promote recruitment of T and NK cells into breast and lung tumors to enhance response^17,19^. Similarly, in lung cancer, SHP2 inhibitors induce CXCR2 ligands that recruit myeloid derived suppressor cells and limit the response to this therapy^18^. Multiple groups including ours have recently demonstrated the involvement of anti-tumor immunity in the therapeutic response to KRAS^G12C^ inhibitors^10,12,13,15^. We recently reported the involvement of adaptive immunity in the therapeutic response of murine lung cancer cells driven by oncogenic EGFR to the TKI, osimertinib^14^. Using scRNA analysis of biopsies from oncogene-defined lung cancers including EGFR mutant and ALK+ cancers, Maynard et al. revealed a transient immunostimulatory effect in lung cancer patients bearing oncogenic RTKs after initial TKI therapy, followed by establishment of an immunosuppressive environment upon progression^33^. These findings are consistent with our data in this study showing that elevated content of immune suppressive neutrophils in pre-treatment ALK+ lung cancer biopsies associates with significantly reduced PFS (Fig. 7). Thus, the present studies add to a growing body of evidence revealing a significant modulatory role for the tumor immune microenvironment on the response of lung tumors to oncogene-targeted drugs.

A major finding of this study is that durable responses to alectinib in the murine EML4-ALK tumor models require a functional adaptive immune system. In the absence of T and B cells, tumors undergo shrinkage, but rapidly rebound even in the continued presence of TKI. Furthermore, differences in the depth and duration of response in the immunocompetent setting correlate with alterations in the TME: increased CD8+ T cells and decreased neutrophils. These data are consistent with our previous report showing that the degree of induction of an IFNγ transcriptional program measured in on-treatment biopsies obtained after ~2 weeks of treatment with EGFR-specific TKIs positively associated with the duration of the therapeutic response^7^. In the Gurule et al. study, a transcriptional signature for T cells was increased in the on-treatment biopsies from patients exhibiting longer treatment duration, suggesting a role for T cells in the duration of response. While we were not able to assess potential variation in duration of treatment among the three murine EML4-ALK immunocompetent models as this would likely require many months of daily oral alectinib therapy in tumor-bearing mice, we postulate that variation in the composition of the TME among individual tumor models may account for the range of therapy durations observed in oncogene-defined subsets of lung cancer patients. This is borne out by data showing a higher neutrophil to lymphocyte ratio is associated with shorter duration of treatment response in ALK patients (Fig. 7).

The results in Figs. 5 and 6 indicate that TME-derived signals and TKI-induced signaling represent distinct inputs into the integrated chemokine expression program that presumably drives immune cell infiltration and content. Notably, EA2 tumors exhibiting a complete therapeutic response to alectinib present with increased baseline expression of the T cell-attracting chemokines, CXCL9 and CXCL10, and CD8+ T cell content at baseline relative to EA1 tumors which exhibit a partial response with viable residual disease. Moreover, EA2 cells exhibit a greater in vitro induction of CXCL10 upon alectinib treatment compared to EA1 cells (Fig. 6) and show a trend for further increases in CD8+ T cell content following alectinib treatment in vivo (Fig. 4). By contrast, CXCL1, CXCL2, CCL2 and TGFB2 with defined pro-tumorigenic roles are more highly expressed by EA1 tumor cells at baseline and may antagonize anti-tumorigenic effects of CD8+ T cells that do infiltrate this model. We propose that differential expression of anti- and pro-tumorigenic chemokines by cancer cells is mediated through inherent differences in the cancer cells, as well as their response to signals emanating from the TME. While these mechanisms are not well understood, difference in signaling pathways, epigenetic regulation of specific chemokine genes and differences in the repertoire of cell surface receptors on distinct cancer cells are likely to dictate the expression pattern of cytokines and chemokines. In a recent report, Tang et al.^18^ demonstrated that treatment of KRAS and EGFR-driven murine lung cancer models with a SHP2 inhibitor increased expression of chemokines that promoted infiltration of T and B cells, but also granulocyte myeloid derived suppressor cells (gMDSC’s) via CXCR1/2 ligands. Combined treatment with SHP2 inhibitor and CXCR1/2 antagonists yielded greater therapeutic benefit compared to SHP2 inhibitor alone. Consistent with this report, we observed higher neutrophils in EA1 tumors, and an inverse relationship between neutrophils and time to progression in ALK+ patients.

The orthotopic implantation model and the panel of murine EML4-ALK cell lines replicate a key feature of the human disease, variable residual disease with continuous alectinib treatment. EA1 and EA3-derived tumors maintain clear residual tumor that can be detected by μCT and drives rapid tumor progression upon termination of therapy. By contrast, EA2 tumors shrink such that no tumor can be visualized with μCT and tumors fail to re-grow upon terminating therapy. Consistent with establishment of immune memory, re-injection of these “alectinib-cured” EA2 tumor-bearing mice with fresh EA2 cells revealed tumor formation in only 1 of 5 mice compared to 5 of 5 mice that propagated tumors following re-injection with a distinct KRAS^G12C^-mutant cell line, LLC (Supplementary Fig. 3b). However, additional studies are needed to confirm the presence of memory T cells. Whether this presentation of therapeutic variation is the result of heterogeneous contribution from host immunity or is mediated by cancer cell autonomous mechanisms, or perhaps both processes, remains a subject for further experimentation. We presume that the rapid outgrowth of all three cell lines when propagated in immunodeficient *nu/nu* mice despite continuous alectinib treatment is mediated by rapid activation of bypass signaling pathways. The observation that outgrowth does not occur in immunocompetent hosts when on TKI treatment suggests that the adaptive immune system actively performs surveillance of the residual tumor cells to block their outgrowth, potentially monitoring additional TKI-induced changes to tumor cells (e.g., chemokines, antigen presentation machinery). Perhaps the initial reduction of cancer cells alters the balance between T cells and cancer cells as proposed in the immunoediting model of cancer^34^ and prevents the ability of cancer cells undergoing bypass signaling to grow. Of note, variant calling algorithms indicate that EA2 cells may bear a threefold higher mutation burden relative to EA1 and EA3 (not shown). Alternatively, it is possible that T cells are capable of negatively regulating bypass signaling mechanisms directly. Additional studies are required to dissect these distinct possibilities.

While the orthotopic implantation model used in these studies exhibits many features of the human disease, there are also limitations. For example, the murine EML4-ALK cell lines and the orthotopic model does not recapitulate the time course of tumor development in patients, and likely does not result in the level of heterogeneity observed in human tumors. In addition, there are likely to be differences in the composition of the TME between murine and human lung tumors. Therefore, future studies using human EML4-ALK tumor cells implanted in humanized mice will be important to confirm our present findings. Finally, extensive analysis of the immune microenvironment in primary human tumor samples obtained from patients exhibiting a range of DepOR and TTP would provide important clinical associations to validate the present findings in murine models.

In conclusion, the murine EML4-ALK cells lines and the orthotopic model are predicted to provide deep insight into the complex regulation of the variable immune microenvironment that is established in individual tumors and serve as a robust platform to test various strategies in experimental therapeutics.

A clinical study reported improved overall survival of EGFR and ALK patients whose tumors presented with high CD8+ T cells prior to initiating TKI therapy^35^. Thus, strategies to increase T cell infiltration and decrease innate immunosuppressive cells in combination with precision oncology agents may represent an approach to improve the depth and duration of response in oncogene-defined subsets of lung cancer patients. Targeting critical chemokines that regulate the recruitment of these populations represents a viable approach.

## Methods

### Analysis of human samples

ALK+ lung cancer patients underwent informed consent for diagnosis and treatment at University of Colorado Hospital. Subsequently, a retrospective chart review was performed using an IRB-approved protocol (Colorado Multi-institutional IRB # 09-0143) allowing for retrospective clincopathologic correlation among the patients. H&E-stained sections of 14 pre-treatment biopsy specimens from ALK+ patients that had been obtained at the time of LUAD diagnosis for routine clinical practice were used. All patients eventually progressed on first-line TKI therapy where PFS ranged from 1 to 54 months (mean and median = 14.2 and 9 months, respectively). Two pathologists independently quantified lymphocytes and polymorphonuclear neutrophils (PMNs) in the specimens and the data were correlated with the time to treatment progression.

### Establishment and culture of murine EML4-ALK cell lines

EA1 and EA3 cells were developed at the University of Colorado Anschutz Medical Center; EA1 cells were previously described as “Y143 cells”^24^. The EML4-ALK cell line referred to herein as EA2 was generously provided by Dr. Andrea Ventura at Memorial Sloan Kettering Cancer Center^23^. To induce primary EML4-ALK tumors, a preparation of recombinant adenovirus encoding Cas9 and gRNAs targeting *Eml4* and *Alk* was purchased from ViraQuest Inc (North Liberty, IA). Tracheal administration of 50 µL Adeno-Cas9-gRNA virus at a dose of 1.5 × 10^8^ PFU/mouse induced multifocal tumor formation in both lungs after 8–12 weeks. Tumors were harvested, minced, and grown in Roswell Park Memorial Institute-1640 (RPMI, Corning) growth media supplemented with 10% fetal bovine serum (FBS, GIBCO) and 1% penicillin–streptomycin (Corning). Cells were maintained in culture and passaged until stable epithelial cell lines were established. Cells were cultured in a humidified incubator with 5% CO_2_ at 37 °C.

### PCR analysis to confirm EML4-ALK expression

To confirm the EML4-ALK inversion and fusion in the cell lines, RNA was isolated using Quick RNA Miniprep kit following manufacturer’s protocol (Zymo Research) and 5 µg of RNA was reverse transcribed using Maxima cDNA Synthesis Kit following manufacturer’s protocol (Fisher Scientific). The resulting cDNA was submitted to PCR analysis using PCRBIO VeriFi^TM^ Mix (PCRBIOSYSTEMS) and previously reported oligonucleotide primers (Eml4-forward: 5’-GAGCCTTGTTGATACATCGTTC-3’ and Alk-reverse: 5’-CAAGGCAGTGAGAACCTGAA-3’^23^). The PCR products (190 bp) were electrophoresed on 2% agarose gel, stained with ethidium bromide, and photographed.

### Immunoblotting

Cells were collected in phosphate-buffered saline, centrifuged, and suspended in lysis buffer (0.5% Triton X-100, 50 mM β-glycerophosphate (pH 7.2), 0.1 mM Na_3_VO_4_, 2 mM MgCl_2_, 1 mM EGTA, 1 mM DTT, 0.3 M NaCl, 2 µg/mL leupeptin and 4 µg/ml aprotinin). Aliquots of the cell lysates containing 50 µg of protein were submitted to SDS-PAGE and immunoblotted for TP53 (Cell Signaling Technology, #32532) and β-actin (Cell Signaling Technology, #4967) as a loading control. Unprocessed and uncropped images of the blot shown in Supplementary Fig. 2c are presented in Supplementary Fig. 5.

### Enzyme-linked immunosorbent assay (ELISA)

Cells were seeded in 10-cm dishes and 24 h later, the cells were treated with 100 nM alectinib or DMSO vehicle control for the indicated times. Conditioned media was collected and assayed for CXCL10/IP-10 with an ELISA kit from Invitrogen and CXCL1, CXCL5/LIX, CCL2 and CCL5/RANTES with ELISA kits from R&D Systems following the manufacturer’s instructions. The measured concentration in each sample was normalized to the total cellular protein per dish and the data are presented as pg/µg protein.

### Cell proliferation assays

Cell lines were plated at 100 cells per well in 96-well tissue culture plates. 24 h later, cells were treated in triplicate with the indicated concentration of alectinib or lorlatinib. Cell number per well by DNA content was determined after 7–10 days of culture using CyQUANT Direct Cell Proliferation Assay (Life Technologies) according to the manufacturer’s instructions. Data are presented as percent of control.

### Mice

Wild-type (WT; C57BL/6J; #000664) and green fluorescent protein (GFP)-expressing mice of C57BL/6 strain (C57BL/6-Tg(UBC-GFP)30Scha/J; #0043530) were obtained from Jackson Laboratory (Bar Harbor, ME). *nu/nu* nude mice (Hsd:Athymic Nude-*Foxn1*^*nu*^**;** #069) were obtained from Envigo (Indianapolis, IN). Experiments were performed in 8-12 week old male and female mice. Animals were bred, housed, and maintained at the University of Colorado Anschutz Medical Campus vivarium. All procedures and manipulations were performed under protocols approved by the Institutional Animal Care and Use Committee at the University of Colorado Anschutz Medical Campus. All methods were carried out in accordance with relevant guidelines and regulations and all methods are reported in accordance with ARRIVE guidelines. Mice were sacrificed using CO_2_ and cervical dislocation as a secondary method.

### Orthotopic mouse model of EML4-ALK lung cancer

All mouse experiments were approved by the Institutional Animal Care and Use Committee at the University of Colorado Anschutz Medical Campus under an approved protocol (Mouse models of Lung Cancer #00148, approved April 15, 2022). Murine lung cancer cells were injected into the left lobe of the lungs of either C57BL/6J mice, *nu/nu* or Rag1^−/−^ mice. Cells were prepared in a solution of 1.35 mg/mL Matrigel (Corning #354234) diluted in Hank’s Balanced Salt Solution (Corning) for injection. Mice were anesthetized with isoflurane, the left side of the mouse was shaved, and a 1 mm incision was made to visualize the ribs and left lobe of the lung. Using a 30-gauge needle, 5 × 10^5^ cells were injected in 40 µL of matrigel cell mix directly into the left lobe of the lung and the incision was closed with staples. Tumors established for 7–10 days and then the mice were submitted to micro-CT (µCT) imaging to obtain pre-treatment tumor volumes. Tumor-bearing mice were randomized into treatment groups (*n* = 10), either 20 mg/kg alectinib or diluent control (H_2_O) by oral gavage 5 days/week until the end of study or termination of treatment. Mice were imaged weekly by µCT imaging to monitor effects of drug treatment on tumor volume. In some experiments, tumor-bearing mice were treated with either H_2_O control or 20 mg/kg alectinib for 4 days. After sacrifice, tumors were measured via digital calipers, and processed for single cell suspensions or formalin fixation.

### Tumor volume quantification

µCT imaging was performed by the Small-Animal IGRT Core at the University of Colorado Anschutz Medical Campus in Aurora, CO using the Precision X-Ray X-Rad 225Cx Micro IGRT and SmART Systems (Precision X-Ray, Madison, CT). Tumor volume was quantified from µCT images using ITK-SNAP software^36^ (www.itksnap.org).

### Tissue harvest and processing

At tissue harvest, lungs were perfused with 5 mL of PBS/heparin (20 U/mL, Sigma) and inflated with 4 mL 4% paraformaldehyde (PFA; Electron Microscopy Scientific). The left lung was removed and fixed for 24 h in 4% PFA, after which they were switched to 70% ethanol. Tissues were processed and embedded into formalin-fixed paraffin-embedded (FFPE) blocks by the Pathology Shared Resource at the University of Colorado Anschutz Medical Campus. Blank slides were cut by the Pathology Shared Resource to be used for multispectral immunofluorescence imaging. To prepare single cell suspensions, mice were sacrificed and the lungs were perfused with 5 mL of PBS/heparin solution. The tumor-bearing left lungs were mechanically dissociated using a razor blade and incubated for 30 min at 37 °C with 3.2 mg/mL Collagenase Type 2 (Worthington, 43C14117B), 0.75 mg/mL Elastase (Worthington, 33S14652), 0.2 mg/mL Soybean Trypsin Inhibitor (Worthington, S9B11099N), and DNAse I 40 µg/mL (Sigma). The resulting single-cell suspensions were filtered through 70 µm strainers (BD Biosciences), washed with FA3 staining buffer [phosphate-buffered saline (PBS) containing 1% FBS, 2 mM EDTA, 10 mM HEPES]. Samples underwent a red blood cell lysis step (0.15 mM NH_**4**_Cl, 10 mM KHCO_**3**_, 0.1 mM Na_**2**_EDTA, pH 7.2), were washed, and filtered through a 40 µm strainer (BD Biosciences). Single-cell suspensions were then submitted to staining for FACS and flow cytometry. For RNAseq of sorted cancer cell suspensions, lungs from 3 to 5 mice were pooled together. For flow cytometry, each data point represents a single-cell suspension of the left lung of one mouse.

### Fluorescence-activated cell sorting (FACS)

Single-cell suspensions were submitted to FACS. Cell sorting was performed at the University of Colorado Cancer Center Flow Cytometry Shared Resource using a MoFlo XDP cell sorter equipped with a 100 micron nozzle (Beckman Coulter). The sorting strategy excluded debris and cell doublets by light scatter and dead cells by DAPI (1 µg/mL). For these studies, lung cancer cells were implanted into GFP+ mice, with cancer cells separated from the host’s GFP-expressing cells by sorting for GFP-negative cells. Immediately after sorting, cells were pelleted and frozen in liquid nitrogen in preparation for RNA extraction. The number of recovered cells ranged from 2.4 × 10^5^ to 15 × 10^5^.

### RNAseq of cancer cells recovered from tumors

Cancer cells were injected into GFP+ mice and recovered from tumors by FACS as previously described^21,37^. Total RNA was isolated from FACS-sorted tumor cells using an RNeasy Plus Mini Kit (QIAgen). The quality and quantity of RNA were analyzed using a bioanalyzer (4150 TapeStation System; Agilent). RNA was submitted to the University of Colorado Cancer Center Genomics Shared Resource for RNAseq library preparation and sequencing using the Illumina HiSEQ2500 (EA1) and NovaSEQ6000 (EA2). Fastq files were analyzed as previously reported^30^. Briefly, the Illumina HiSeq Analysis Pipeline was followed. Reads were quality checked using FastQC, aligned with TopHatv2 using the *Mus musculus* mm10 reference genome (UC Santa Cruz), and then the aligned reads were assembled into transcripts using Cufflinks v2.0.2 to estimate their abundance. Data are shown as fragments per kilobase of exon per million fragments mapped (FPKM).

### RNAseq of murine EML4-ALK cell lines

The murine EML4-ALK cell lines cultured in 10 cm dishes were treated for 1–5 days with 0.1% DMSO or 100 nM alectinib. RNA was submitted to the University of Colorado Cancer Center Genomics Shared Resource where libraries were generated and sequenced on the NovaSeq 4000 to generate 2 × 151 reads. Fastq files were quality checked with FastQC, Illumina adapters trimmed with bbduk, and mapped to the mouse mm10 genome with STAR aligner. Counts were generated by STAR’s internal counter and reads were normalized to counts per million reads mapped (CPM) using the edgeR R package^38^. Heatmaps were generated in Prism 9 (GraphPad Software, San Diego, CA).

### Multispectral imaging

Methods for multispectral imaging have previously been published and the methods herein are described in brief^33,39^. Following the manufacturer’s protocol, FFPE slides were sequentially stained using Opal IHC Multiplex Assay (PerkinElmer, Waltham, MA) by the Human Immune Monitoring Shared Resource at the University of Colorado Anschutz Medical Campus. The Vectra Polaris Imaging System (PerkinElmer) scanned the whole slide. The panel used for the Vectra Polaris were as follows in sequential order: CD11b, CD64, EpCAM. CD11c, B220, CD8, F4/80. Approximately 5 regions of interest (ROIs) were evaluated per tumor. Images were analyzed using inForm Tissue Analysis Software (v2.4.8, Akoya, Menlo Park, CA) to un-mix fluorochromes, remove autofluorescence, segment tissue and cells, and phenotype cells. Data analysis was performed as previously described by our group^21,39^. Briefly, data acquired from inForm was analyzed using the R package Akoya Biosciences phenoptrReports. The count_phenotypes function was used to aggregate phenotype counts for each slide. Data are presented as cell counts.

### Flow cytometry

The single-cell suspension samples were blocked in anti-mouse CD16/CD32 (clone 93; eBioscience) at 1:200 on a rocker for 15 min at 4 °C. Next, fix viability dye (LIVE/DEAD Fixable Aqua Dead Cell Stain Kit; 1:200; Invitrogen) and conjugated antibodies were added (see below) to the single cell suspension. Cells were incubated in the dark at 4 °C for 60 min. Cells were then resuspended in FA3 buffer and ran on the Gallios Flow Cytometer (Beckman Coulter). For compensation, single-stained beads (VersaComp Antibody Capture Bead Kit; Beckman Coulter) and a single-stained cell-mix of all samples analyzed were used. Flow cytometry was analyzed using Kaluza Analysis Software (v2.0, Beckman Coulter). Compensation was first performed on the single-stained bead controls and then confirmed using the single-stained cell mixture.

### Antibody panel

CD11b-FITC (clone M1/70; 1:100; BioLegend), CD64-PE (clone X54-5/7.1; 1:100; BD Biosciences), MHCII-Dazzle (clone M5/111.15.2; 1:250; BioLegend), Ly6C-PerCP/Cy5.5 (clone HK1.4; 1:100; BioLegend), Ly6G-PE/Cy7 (clone 1A8; 1:200; BioLegend), SigF-A647 (clone E50-2440; 1:100; BD Biosciences), CD45-AF700 (clone 30-F11; 1:100; Invitrogen), CD11c-APC/Cy7 (clone HL3; 1:100; BD Biosciences), MHCI-eF450 (clone 28-14-8; 1:100; Invitrogen), CD4-V500 (clone RM4-5; 1:200; BD Biosciences; used only for compensation)

### Graphical analysis and statistics

Graphing and statistical analysis was performed using GraphPad Prism version 9.2.0 (San Diego, CA) or R version 4.0.3, R studio (for multispectral imaging analyses). To determine significance between groups, Student’s *t* tests or one-way ANOVA tests were performed. Data are presented as mean ± SEM. Significant differences are indicated by *p < 0.05, **p < 0.01, ***p < 0.001, or ****p < 0.0001.

### Reporting summary

Further information on research design is available in the Nature Research Reporting Summary linked to this article.

## Supplementary information

Supplemental Information

REPORTING SUMMARY

## Data Availability

The RNAseq data generated in this study are deposited in GEO Datasets (GSE204918 and GSE217115).
