# Supplementary material for: Durable responses to alectinib in murine models of EML4-ALK lung cancer requires adaptive immunity
Source: NPJ Precis Oncol. 2023 Feb 4;7:15. doi: 10.1038/s41698-023-00355-2 (PMC9899278; doi:10.1038/s41698-023-00355-2)
Supplement: Supplementary file 1 — Supplemental Information [file 41698_2023_355_MOESM1_ESM.pdf]

Supplementary Information

Durable responses to alectinib in murine models of EML4-ALK lung cancer requires adaptive immunity

Emily K. Kleczko[1], Trista K. Hinz[2,5], Teresa T. Nguyen[2], Natalia J. Gurule[2], Andre Navarro[1], Anh T. Le[1], Amber M. Johnson[1], Jeff Kwak[1], Diana I. Polhac[2], Eric T. Clambey[3], Mary Weiser-Evans[1], Daniel T. Merrick[4], Michael C. Yang[4], Tejas Patil[1], Erin L. Schenk[1,*], Lynn E. Heasley[2,5,*], Raphael A. Nemenoff[1,*]

[1] Departments of Medicine, [2]Craniofacial Biology, [3]Anesthesiology and Pathology[4], University of Colorado Anschutz Medical Campus, Aurora, CO, USA

[5]Eastern Colorado VA Healthcare System, Rocky Mountain Regional VA Medical Center, Aurora, Colorado, USA

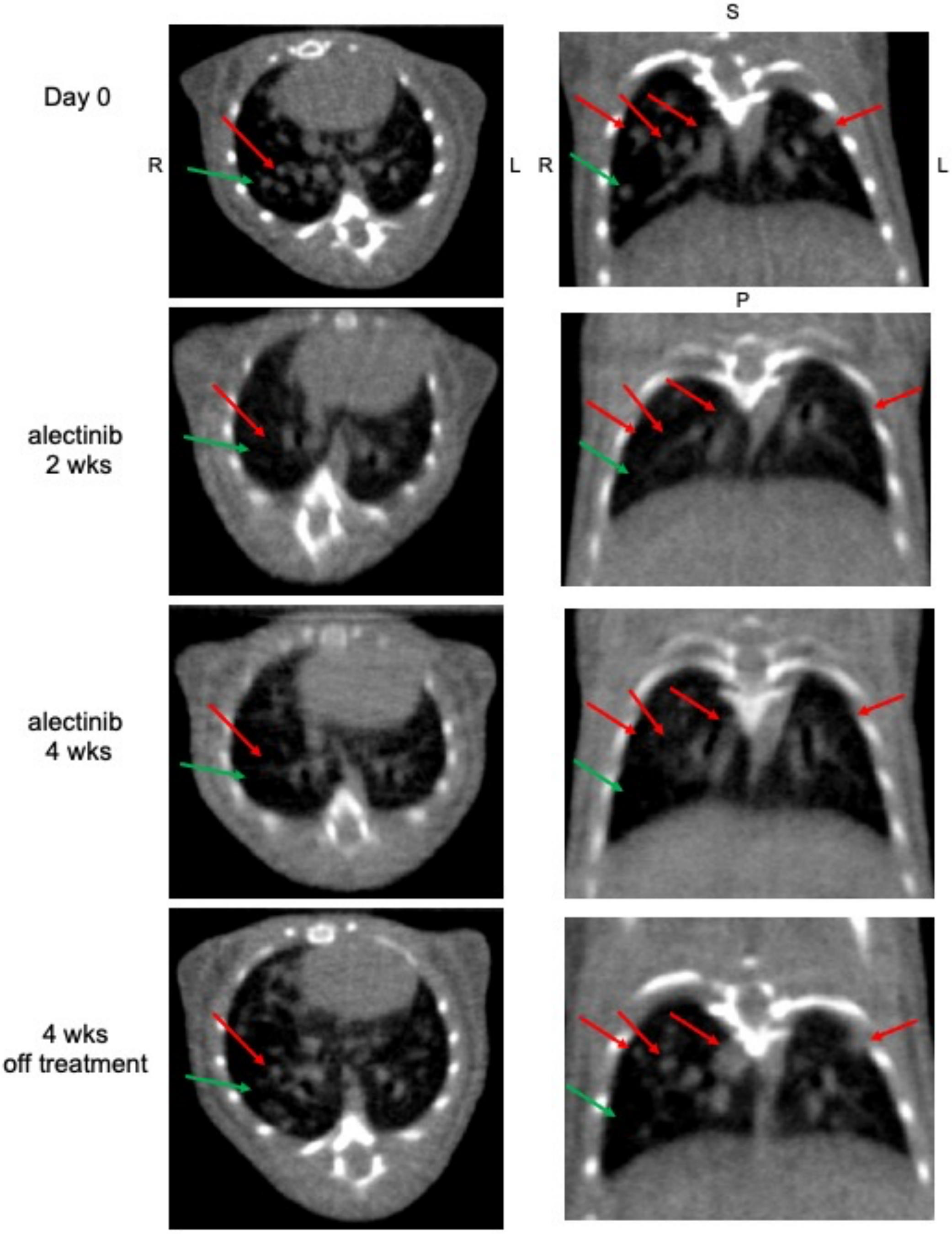

Supplementary Figure 1. Alectinib sensitivity of primary murine EML4-ALK tumors. Recombinant adenoviruses encoding Cas9 and gRNAs targeting *Eml4* and *Alk* were instilled intratracheally into C57BL/6 mice. The mice were routinely monitored by μCT for emergence of lung tumors and after 8 weeks (Day 0 in figure), mice were submitted to daily oral gavage with alectinib (20 mg/kg) and μCT

continued on a weekly basis. Following 4 weeks of alectinib treatment, therapy was terminated and μCT imaging continued. Serial images of an alectinib-treated mouse (representative of 4 others) are shown. R = right, L = left, S = superior, P = posterior. Red arrows identify lesions that shrank upon alectinib treatment and subsequently re-grew following therapy termination. Green arrows identify lesions that shrank with treatment and did not regrow upon termination of alectinib therapy.

a

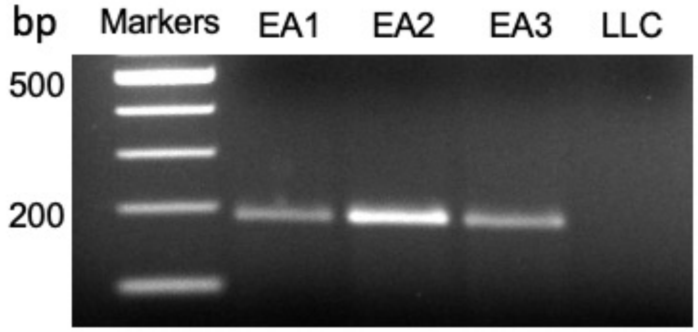

b

	RNA expression, CPM			
Gene	EA1	EA2	EA3	LLC
ALK	646	1204	700	0
MAPK1	1756	2602	1577	1664

c

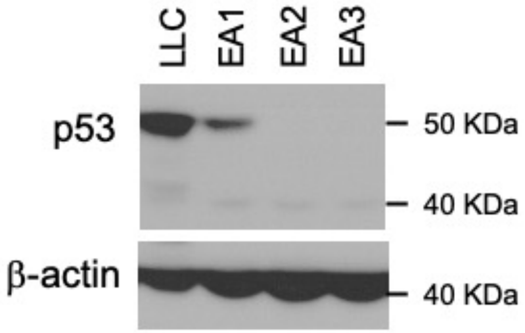

Supplementary Figure 2. RNA expression of EML4-ALK in murine cell lines. a, RNA was purified from EA1, EA2 and EA3 cells and submitted to reverse transcription PCR to amplify the coding sequences expressed from the rearranged EML4-ALK gene fusion. **b**, RNAseq data from the cell lines was queried for ALK mRNA expression with MAPK1 as a control house-keeping gene. The LLC cell line is a KRAS mutant line that does not express ALK. **c**, Cell extracts were prepared from the indicated murine cell lines and submitted to immunoblot analysis for TP53 and β-actin as a loading control. LLC cells bear a missense mutation in TP53, EA1 was developed in TP53 wild-type mice and EA2 and EA3 were generated in TP53 null mice.

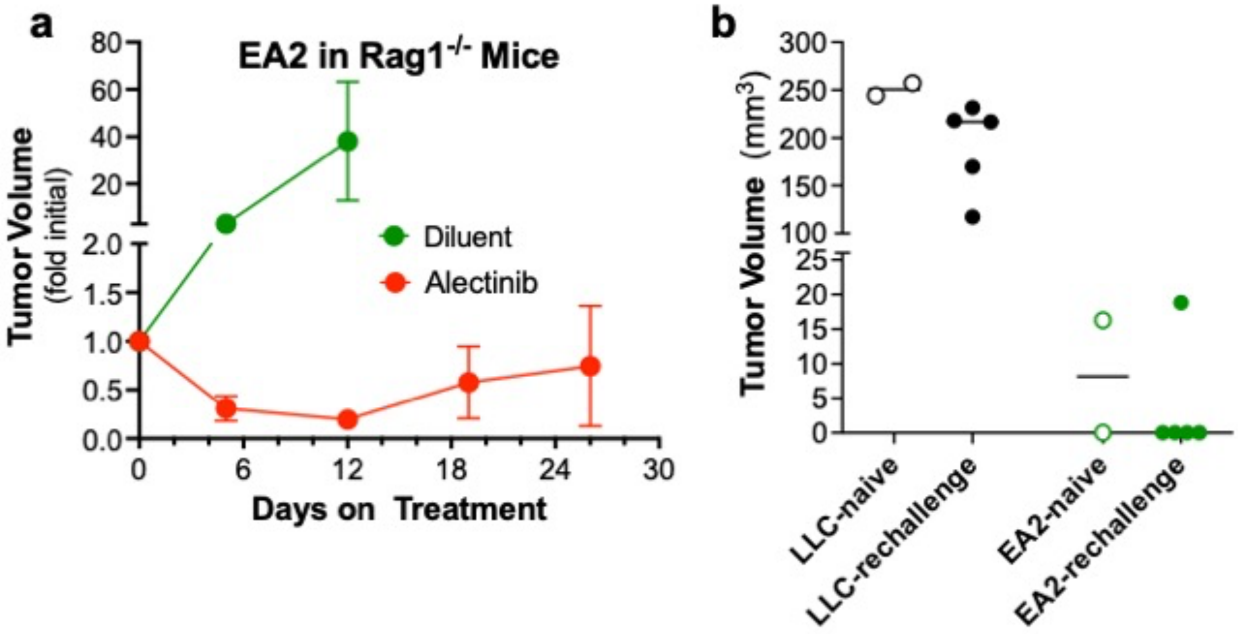

Supplementary Figure 3. Lack of alectinib-treatment durability in EA2-bearing Rag1-/- mice and evidence for immunologic memory following alectinib-induced EA2 tumor elimination. a, EA2 cells (500,000 cells/mouse) were inoculated into the left lungs of Rag1$^{-/-}$ mice and tumors were allowed to establish for 2 weeks. A pre-treatment μCT image was acquired, the mice were randomized into treatment groups (n=8) and treated daily by oral gavage with diluent control or alectinib (20 mg/kg). Tumor volume was monitored by serial μCT and the average tumor volumes (means and SEM) presented as fold-change from initial volumes. **b**, EA2 cells (500,000 cells/mouse) were inoculated into the left lungs of C57BL/6 mice and tumors were allowed to establish for 10 days. Pre-treatment μCT images were obtained and the mice were treated with alectinib (20 mg/kg) daily for 31 days at which point no lung tumors were detectable by μCT imaging. Treatment was terminated and 44 days later, 500,000 LLC cells (n=5) or EA2 cells (n=5) were injected into the right flanks of the EA2 tumor-bearing and treated mice (rechallenge) as well as naïve mice (n=2 for each cell line). The tumor volumes of the flank tumors was monitored with calipers for 3 (LLC tumors) and 4 (EA2 tumors) weeks, respectively.

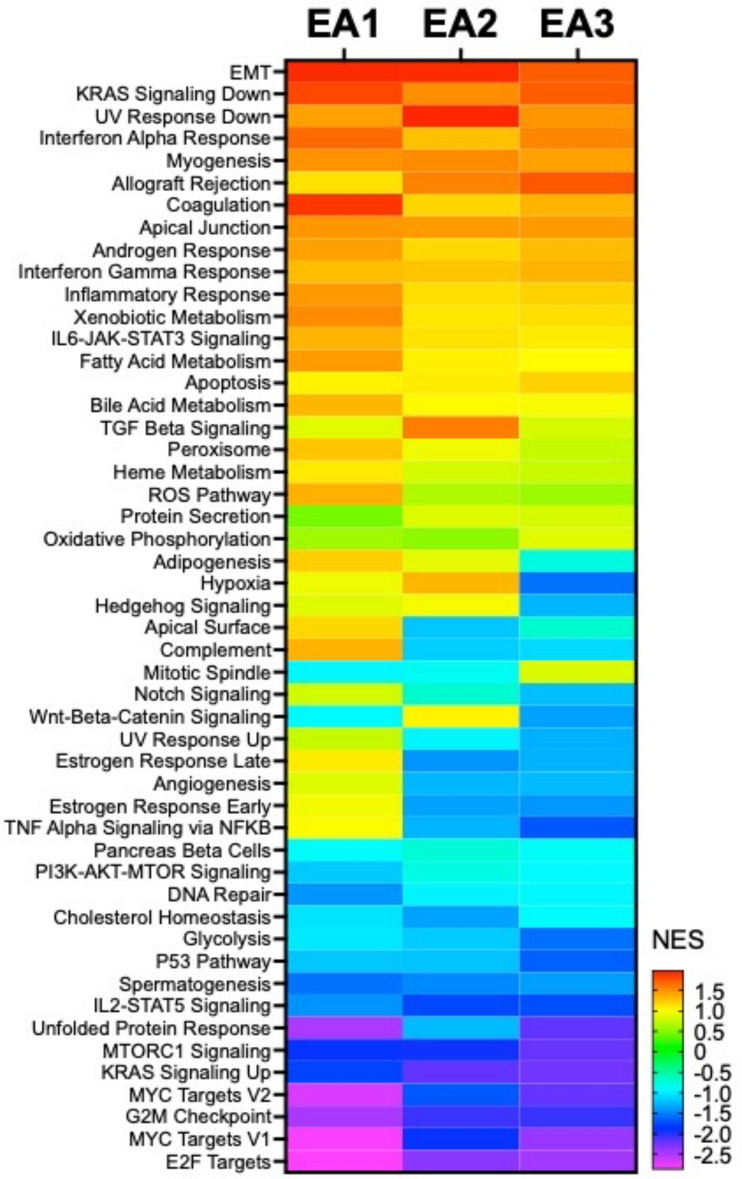

Supplementary Figure 4. Gene-set enrichment analysis of RNAseq data from murine EML4-ALK cell lines treated *in vitro* with alectinib. RNA was purified from EA1, EA2 and EA3 cells treated *in vitro* with DMSO or alectinib (100 nM) for 1-5 days and submitted for RNAseq. For this analysis, the different time points were considered as replicates (n=4) and DMSO vs. alectinib-treated samples analyzed with GSEA using the Hallmark Pathways. The heatmap presents the normalized enrichment scores (NES) in the alectinib-treated samples (see color bar for relative scores).

a

b

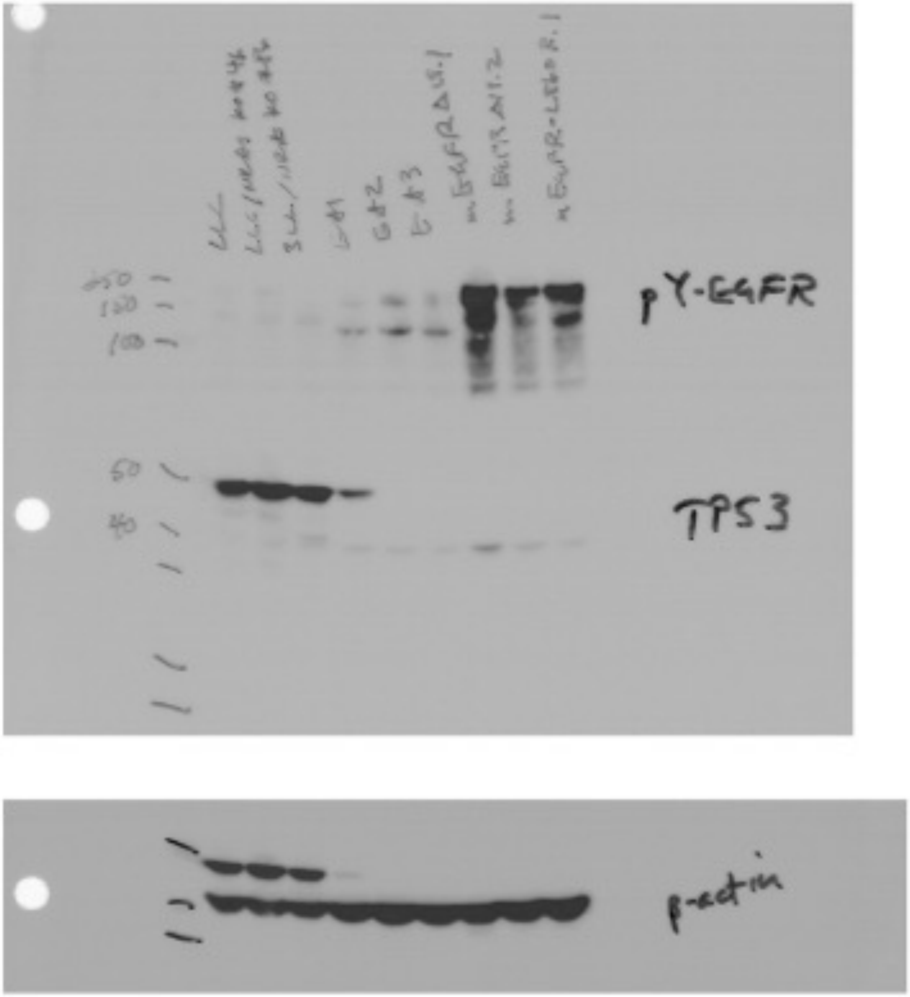

Supplementary Figure 5. Uncropped and unprocessed scans of immunoblots in Supplementary Figure 2c. In **a**, the indicated samples were probed for phospho-tyrosine EGFR (top portion of blot) or murine TP53 (bottom portion of blot). In **b**, the bottom portion of the blot was reprobed for b-actin. Residual TP53 antibody remained after stripping and accounts for the top band in the first 4 lanes.
