# Supplementary material for: Durable responses to alectinib in murine models of EML4-ALK lung cancer requires adaptive immunity
Source: NPJ Precis Oncol. 2023 Feb 4;7:15. doi: 10.1038/s41698-023-00355-2 (PMC9899278; doi:10.1038/s41698-023-00355-2)
Supplement: Supplementary file 2 — REPORTING SUMMARY [file 41698_2023_355_MOESM2_ESM.pdf]

nature portfolio

Corresponding author(s): Raphael A. Nemenoff, Lynn E. Heasley, Erin L. Schenk

Last updated by author(s): 2023/01/03

Reporting Summary

Nature Portfolio wishes to improve the reproducibility of the work that we publish. This form provides structure for consistency and transparency in reporting. For further information on Nature Portfolio policies, see our Editorial Policies and the Editorial Policy Checklist.

Statistics

For all statistical analyses, confirm that the following items are present in the figure legend, table legend, main text, or Methods section.

n/a	Confirmed	
☐	☒	The exact sample size (*n*) for each experimental group/condition, given as a discrete number and unit of measurement
☐	☒	A statement on whether measurements were taken from distinct samples or whether the same sample was measured repeatedly
☐	☒	The statistical test(s) used AND whether they are one- or two-sided<br>*Only common tests should be described solely by name; describe more complex techniques in the Methods section.*
☒	☐	A description of all covariates tested
☐	☒	A description of any assumptions or corrections, such as tests of normality and adjustment for multiple comparisons
☐	☒	A full description of the statistical parameters including central tendency (e.g. means) or other basic estimates (e.g. regression coefficient) AND variation (e.g. standard deviation) or associated estimates of uncertainty (e.g. confidence intervals)
☒	☐	For null hypothesis testing, the test statistic (e.g. *F*, *t*, *r*) with confidence intervals, effect sizes, degrees of freedom and *P* value noted<br>*Give P values as exact values whenever suitable.*
☒	☐	For Bayesian analysis, information on the choice of priors and Markov chain Monte Carlo settings
☒	☐	For hierarchical and complex designs, identification of the appropriate level for tests and full reporting of outcomes
☒	☐	Estimates of effect sizes (e.g. Cohen's *d*, Pearson's *r*), indicating how they were calculated

Our web collection on statistics for biologists contains articles on many of the points above.

Software and code

Policy information about availability of computer code

Data collection	No software or code was used for data collection.
Data analysis	Fastq files were quality checked with FastQC, Illumina adapters trimmed with bbduk, and mapped to the mouse mm10 genome with STAR aligner. Counts were generated by STAR's internal counter and reads were normalized to counts per million reads mapped (CPM) using the edgeR R package

For manuscripts utilizing custom algorithms or software that are central to the research but not yet described in published literature, software must be made available to editors and reviewers. We strongly encourage code deposition in a community repository (e.g. GitHub). See the Nature Portfolio guidelines for submitting code & software for further information.

Data

Policy information about availability of data

All manuscripts must include a data availability statement. This statement should provide the following information, where applicable:

- Accession codes, unique identifiers, or web links for publicly available datasets
- A description of any restrictions on data availability
- For clinical datasets or third party data, please ensure that the statement adheres to our policy

The RNAseq data generated in this study have been deposited in the GEO Datasets (GSE204918 and GSE217115).

Human research participants

Policy information about studies involving human research participants and Sex and Gender in Research.

Reporting on sex and gender	*Use the terms sex (biological attribute) and gender (shaped by social and cultural circumstances) carefully in order to avoid confusing both terms. Indicate if findings apply to only one sex or gender; describe whether sex and gender were considered in study design whether sex and/or gender was determined based on self-reporting or assigned and methods used. Provide in the source data disaggregated sex and gender data where this information has been collected, and consent has been obtained for sharing of individual-level data; provide overall numbers in this Reporting Summary. Please state if this information has not been collected. Report sex- and gender-based analyses where performed, justify reasons for lack of sex- and gender-based analysis.*
Population characteristics	*Describe the covariate-relevant population characteristics of the human research participants (e.g. age, genotypic information, past and current diagnosis and treatment categories). If you filled out the behavioural & social sciences study design questions and have nothing to add here, write "See above."*
Recruitment	*Describe how participants were recruited. Outline any potential self-selection bias or other biases that may be present and how these are likely to impact results.*
Ethics oversight	*Identify the organization(s) that approved the study protocol.*

Note that full information on the approval of the study protocol must also be provided in the manuscript.

Field-specific reporting

Please select the one below that is the best fit for your research. If you are not sure, read the appropriate sections before making your selection.

☒ Life sciences ☐ Behavioural & social sciences ☐ Ecological, evolutionary & environmental sciences

For a reference copy of the document with all sections, see nature.com/documents/nr-reporting-summary-flat.pdf

Life sciences study design

All studies must disclose on these points even when the disclosure is negative.

Sample size	For animal experiments sample was size was determined by a power calculation with n=10 being used.
Data exclusions	No data was excluded.
Replication	All attempts at replication were successful..
Randomization	Tumor bearing mice were randomly assigned to treatment groups.
Blinding	Invesigators were blinded for immunostaining and quantification of tumor size.

Reporting for specific materials, systems and methods

We require information from authors about some types of materials, experimental systems and methods used in many studies. Here, indicate whether each material, system or method listed is relevant to your study. If you are not sure if a list item applies to your research, read the appropriate section before selecting a response.

Materials & experimental systems

n/a	Involved in the study	
☐	☒	Antibodies
☐	☒	Eukaryotic cell lines
☒	☐	Palaeontology and archaeology
☐	☒	Animals and other organisms
☐	☒	Clinical data
☒	☐	Dual use research of concern

Methods

n/a	Involved in the study	
☒	☐	ChIP-seq
☐	☒	Flow cytometry
☒	☐	MRI-based neuroimaging

Antibodies

Antibodies used	Antibody Panel: CD11b-FITC (clone M1/70; 1:100; BioLegend), CD64-PE (clone X54-5/7.1; 1:100; BD Biosciences), MHCII-Dazzle (clone M5/111.15.2; 1:250; BioLegend), Ly6C-PerCP/Cy5.5 (clone HK1.4; 1:100; BioLegend), Ly6G-PE/Cy7 (clone 1A8; 1:200; BioLegend), SigF-A647 (clone E50-2440; 1:100; BD Biosciences), CD45-AF700 (clone 30-F11; 1:100; Invitrogen), CD11c-APC/Cy7 (clone HL3; 1:100; BD Biosciences), MHCI-eF450 (clone 28-14-8; 1:100; Invitrogen), CD4-V500 (clone RM4-5; 1:200; BD Biosciences; used only for compensation)
Validation	All antibodies were from commercial suppliers and had been validated by the company. For immunoblotting, control samples with normal IgG were run.

Eukaryotic cell lines

Policy information about cell lines and Sex and Gender in Research

Cell line source(s)	All cell lines were derived from tumors in C57BL/6 mice.
Authentication	Cell lines were authenticated by determining in vitro response to alectinib, and expression of the EML4/ALK fusion protein.
Mycoplasma contamination	All cell lines were regularly tested for mycoplasma.
Commonly misidentified lines (See ICLAC register)	None

Animals and other research organisms

Policy information about studies involving animals; ARRIVE guidelines recommended for reporting animal research, and Sex and Gender in Research

Laboratory animals	C57BL/6 mice, both male and female ages 6-10 weeks.
Wild animals	None
Reporting on sex	Both male and female mice were used, and no sex-specific differences were observed in tumor growht.
Field-collected samples	N/A
Ethics oversight	No ethics approval was required.

Note that full information on the approval of the study protocol must also be provided in the manuscript.

Clinical data

Policy information about clinical studies
All manuscripts should comply with the ICMJE guidelines for publication of clinical research and a completed CONSORT checklist must be included with all submissions.

Clinical trial registration	N/A
Study protocol	N/A
Data collection	N/A
Outcomes	N/A

Flow Cytometry

Plots

Confirm that:

☒ The axis labels state the marker and fluorochrome used (e.g. CD4-FITC).

☒ The axis scales are clearly visible. Include numbers along axes only for bottom left plot of group (a 'group' is an analysis of identical markers).

☐ All plots are contour plots with outliers or pseudocolor plots.

☒ A numerical value for number of cells or percentage (with statistics) is provided.

Methodology

Sample preparation	The tumor-bearing left lungs were mechanically dissociated using a razor blade and incubated for 30 minutes at 37°C with 3.2mg/mL Collagenase Type 2 (Worthington, 43C14117B), 0.75 mg/ml Elastase (Worthington, 33S14652), 0.2 mg/ml Soybean Trypsin Inhibitor (Worthington, S9B11099N), and DNAse I 40 µg/ml (Sigma). The resulting single cell suspensions were filtered through 70µm strainers (BD Biosciences), washed with FA3 staining buffer [phosphate-buffered saline (PBS) containing 1% FBS, 2mM EDTA, 10mM HEPES]. Samples underwent a red blood cell lysis step (0.15 mM NH_4Cl, 10 mM $KHCO_3$, 0.1 mM Na_2EDTA, pH 7.2), were washed, and filtered through a 40 µm strainer (BD Biosciences).
Instrument	Gallios Flow Cytometer (Beckman Coulter)
Software	For compensation, single-stained beads (VersaComp Antibody Capture Bead Kit; Beckman Coulter) and a single-stained cell-mix of all samples analyzed were used. Flow cytometry was analyzed using Kaluza Analysis Software (v2.0, Beckman Coulter). Compensation was first performed on the single-stained bead controls and then confirmed using the single-stained cell mixture.
Cell population abundance	The cell population abundance is described in References 22 and 26 from our lab, which are cited in the manuscript.
Gating strategy	The gating strategy is identical to that published by our lab in References 22 and 26 from our lab which are cited in the manuscript.

☐ Tick this box to confirm that a figure exemplifying the gating strategy is provided in the Supplementary Information.
